# Sensor Data Analytics: Challenges and Methods for Data-Intensive Applications

**DOI:** 10.3390/e24070850

**Published:** 2022-06-21

**Authors:** Felipe Ortega, Emilio L. Cano

**Affiliations:** Data Science Laboratory, Research Centre for Intelligent Information Technologies, Rey Juan Carlos University, 28933 Madrid, Spain

Sensors have become a key element for the development of the Information Society. An ever-increasing number of improved sensor devices capture information for decision-making tools, either to be interpreted by humans or to be plugged back into the system for autonomous operation, self-diagnostics and resilience. It is possible to find sensor applications spanning almost any area, including healthcare and medicine, retail and logistics, smart agriculture and animal farming, industry digitalisation, smart cities, energy grids, transport or security, among many others [1]. Analytics is a term connected to the practice of data science that refers to the analysis of data using statistical tools and techniques, machine learning, information theory, pattern recognition and other methods. Outcomes stemming from this task constitute essential inputs for data-driven decision-making [2,3].

The current overabundance of data, generated in many cases by sensors, together with the refinement of standard methodologies for data science and engineering [4] has led to the rise of a fourth scientific paradigm, the so-called *data-intensive scientific discovery* [5]. Indeed, one of the most challenging aspects for the development of data-intensive applications has been how to cope with massive and complex datasets effectively, especially in situations in which real-time requirements arise [6,7]. In this regard, sensors provide an unrivalled data source to match these needs, as they can provide timestamped information with enough level of detail to characterise observed phenomena adequately.

Information theory [8] plays a central role for knowledge extraction in sensor data analytics, such as the analysis of data in the frequency domain [9], the essential concept of *entropy* [10] and efficient data representation and compression [11]. As a result, many new methods based on information theory have been developed in modern data science [12]. This Special Issue presents nine original contributions encompassing a wide variety of sensor data analytics applications, in which information theory is used to obtain knowledge from data in different domains.

Gajowniczek et al. [13] develop a novel method for data streams clustering, applicable to complete time series representing customer electricity consumption. This method leverages new Fast Fourier Transform (FFT) [9] features to improve its performance, showing the importance of information theory principles in this type of analysis. Wearable sensors tracking human activity and behaviour are at the core of several works, including applications in rehabilitation of visually impaired people [14], automated human activity recognition [15] and walking behaviour detection for elderly people [16]. The last two works attach importance to the application of information gain and neural networks to detect activity profiles accurately. Alfaro et al. [17] propose a new method to distribute the training process using the SVM algorithm, which can be applicable to Wireless Sensor Networks (WSN), aggregating the local contributions from individual sensors using Voronoi regions. Once again, this demonstrates the critical role of information aggregation in this kind of energy and location-aware sensor application. Sensor placement optimisation is the topic of another work [18], using Gaussian priors and the Fisher Information Matrix (FIM) to show important properties that can enhance recommendations on the best possible location for a given device set. Sun et al. present an interesting application of sensor data analytics to estimate vehicles accident risk [19]. This is an emerging topic that raises significant interest among insurance companies, taking advantage of the more precise tracking capabilities enabled by built-in sensors installed in vehicles. In turn, Esteban-Escaño et al. present an interesting application of sensor data analysis to predict acidemia in electronic fetal monitoring [20], using machine learning algorithms, stressing the use of cross-entropy optimisation function along this process, to adjust the best possible predictive model. Finally, the last work [21] presents a novel methodology for cattle behaviour profiling and classification that, again, uses both time-domain and frequency-domain features to improve the accuracy of this classification task.

In summary, these contributions offer a diverse and representative portfolio of sensor data analytics applications in different scenarios, in which information theory and data science methods perform a central role in order to successfully accomplish the proposed challenges in each case.
